# High site fidelity and restricted ranging patterns in southern Australian bottlenose dolphins

**DOI:** 10.1002/ece3.3674

**Published:** 2017-11-26

**Authors:** Cecilia Passadore, Luciana Möller, Fernando Diaz‐Aguirre, Guido J. Parra

**Affiliations:** ^1^ Cetacean Ecology, Behaviour and Evolution Lab College of Science and Engineering Flinders University Bedford Park SA Australia; ^2^ Molecular Ecology Lab College of Science and Engineering Flinders University Bedford Park SA Australia

**Keywords:** delphinids, estuary, inshore, site fidelity, spatial ecology, *Tursiops* cf. *australis*, utilization distribution

## Abstract

Information on site fidelity and ranging patterns of wild animals is critical to understand how they use their environment and guide conservation and management strategies. Delphinids show a wide variety of site fidelity and ranging patterns. Between September 2013 and October 2015, we used boat‐based surveys, photographic identification, biopsy sampling, clustering analysis, and geographic information systems to determine the site‐fidelity patterns and representative ranges of southern Australian bottlenose dolphins (*Tursiops* cf. *australis*) inhabiting the inner area of Coffin Bay, a highly productive inverse estuary located within Thorny Passage Marine Park, South Australia. Agglomerative hierarchical clustering (AHC) of individuals’ site‐fidelity index and sighting rates indicated that the majority of dolphins within the inner area of Coffin Bay are “regular residents” (*n* = 125), followed by “occasional residents” (*n* = 28), and “occasional visitors” (*n* = 26). The low standard distance deviation indicated that resident dolphins remained close to their main center of use (range = 0.7–4.7 km, X ± *SD* = 2.3 ± 0.9 km). Representative ranges of resident dolphins were small (range = 3.9–33.5 km^2^, X ± *SD* = 15.2 ± 6.8 km^2^), with no significant differences between males and females (Kruskal–Wallis, χ^2^ = 0.426, *p *=* *.808). The representative range of 56% of the resident dolphins was restricted to a particular bay within the study area. The strong site fidelity and restricted ranging patterns among individuals could be linked to the high population density of this species in the inner area of Coffin Bay, coupled with differences in social structure and feeding habits. Our results emphasize the importance of productive habitats as a major factor driving site fidelity and restricted movement patterns in highly mobile marine mammals and the high conservation value of the inner area of Coffin Bay for southern Australian bottlenose dolphins.

## 1 INTRODUCTION

Movement and space use patterns of individual animals affect population distribution and abundance, habitat selection, species interactions, and social and population structure, which in turn influence individuals’ fitness (Börger, 2016; Nathan et al., 2008). Studies on multiple taxa have shown that the ranging patterns of individuals (i.e., location and area used within a study site) and the tendency of animals to remain in the same area or return to it multiple times (i.e., site fidelity, Switzer, 1993; White & Garrott, 2012) are driven by changes in individual's needs and the distribution of its conspecifics, predators, and resources (Nathan et al., 2008; Switzer, 1993, 1997). In low‐productive landscapes/seascapes with heterogeneous habitats, individuals improve their fitness by following an opportunistic strategy of accessing the highest quality habitats available, which result in animals showing low site fidelity and ranging across large areas (Edwards, Nagy, & Derocher, 2009; Silva et al., 2008). By contrast, in landscapes/seascapes where high‐quality habitats are available and resources are predictable, individuals can develop high site fidelity and range in relatively small areas (Habel, Hillen, Schmitt, & Fischer, 2016; Knip, Heupel, & Simpfendorfer, 2012). Such patterns of site fidelity and space use have important implications for the conservation of animals. For example, species with high site fidelity and restricted ranging patterns are more prone to population declines due to local threats such as habitat degradation and loss (Warkentin & Hernández, 1996), and human‐caused mortalities (e.g., due to bycatch, Atkins et al., 2016). Therefore, understanding animal patterns of site fidelity and space use is fundamental for assessing the effects of human impacts and to guide conservation and management strategies.

Marine mammals such as dolphins live in fluid, open environments with few boundaries, feed on mobile prey, and have low transport costs per unit weight (Williams, 1999). As a result, they are highly mobile and tend to have larger home ranges than terrestrial mammals of similar size (Tucker, Ord, & Rogers, 2014). Delphinids show a wide variety of site fidelity and ranging patterns. Some individuals may occupy large ranges while others are restricted to smaller areas; some display year‐round residency patterns while others are seasonal or transient visitors (e.g., Connor, Wells, Mann, & Read, 2000; Hunt et al., 2017; McGuire & Henningsen, 2007; Parra, Corkeron, & Marsh, 2006; Silva et al., 2008; Zanardo, Parra, & Möller, 2016). This variety of site fidelity and ranging patterns is thought to be mainly linked to the spatial and temporal predictability of available food resources (Gowans, Würsig, & Karczmarski, 2008). The socioecological model proposed by Gowans et al. (2008) for delphinids predicts that in areas with predictable resources, dolphins should remain resident, range over relatively small areas, and form small groups to reduce intraspecific competition for food. In contrast, when resources vary in space and time, dolphins should be more transient, range widely to access sufficient resources, and form larger groups to increase foraging success and reduce predation risk (Gowans et al., 2008).

Other factors known to influence dolphin site fidelity and ranging patterns include age and sex. In some populations of bottlenose dolphins (*Tursiops* spp.), adult females display smaller ranging patterns than adult males (Möller, 2012; Sprogis, Raudino, Rankin, MacLeod, & Bejder, 2016; Urian, Hofmann, Wells, & Read, 2009; Wells et al., 2017), while both sexes show similar ranging patterns during the juvenile period (McHugh, Allen, Barleycorn, & Wells, 2011). Sex‐biased dispersal in adult dolphins is typical of mammals with polygynous mating systems, where males tend to range over larger areas to increase mating opportunities with reproductive females, while females tend to be more philopatric to their natal area (Möller & Beheregaray, 2004; Sprogis et al., 2016). In populations of bottlenose dolphins where both sexes exhibit a high degree of philopatry to natal areas, fitness benefits related to familiarity with associates and foraging habitats may explain such patterns, with reduced mother–offspring association after weaning diminishing mother–son inbreeding and mother–daughter resource competition (Tsai & Mann, 2013).

**Figure 1:**
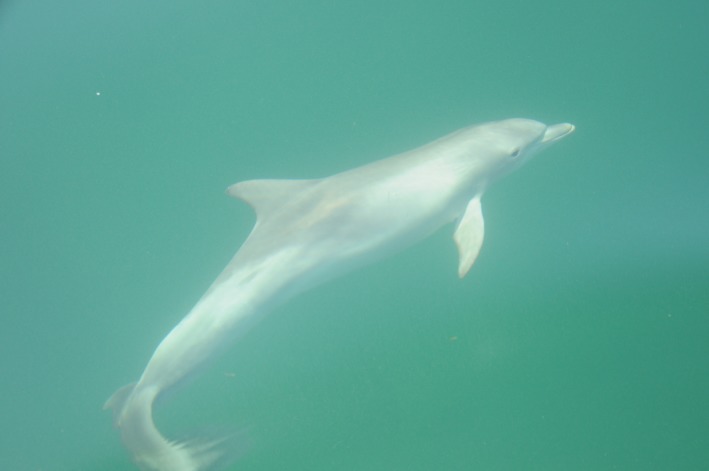
Southern Australian bottlenose dolphin (*Tursiops* cf. *australis*)

Bottlenose dolphins are found throughout coastal and inshore waters of Australia (Leatherwood & Reeves, 2012). A new species, endemic to southeastern and southern Australia, the Burrunan dolphin (*Tursiops australis*), was recently described (Charlton‐Robb et al., 2011). Their taxonomic status, however, is not fully accepted (Committee on Taxonomy 2016; Perrin, Rosel, & Cipriano, 2013), and thus, we refer to them here as southern Australian bottlenose dolphins (*Tursiops* cf. *australis*; Figure 1). Only two small resident populations of southern Australian bottlenose dolphins are known to occur in Victoria (Charlton‐Robb, Taylor, & McKechnie, 2015), while recent studies indicate that this species is relatively abundant in South Australia (Passadore, Möller, Diaz‐Aguirre, & Parra, 2017; Zanardo et al., 2016). Capture–recapture modeling of photographic‐identification (photo‐ID) data and molecular analyses of biopsy samples collected in the inner area of Coffin Bay, an inverse estuary located in temperate waters of a multiple‐use marine park in South Australia, indicated that this area offers highly favorable habitat for both males and females of this species (Passadore et al., 2017). The demography of southern Australian bottlenose dolphins in the inner area of Coffin Bay is characterized by high year‐round abundance (265; 95% CI: 253–278), and low temporary emigration rates (0.02; 95% CI: 0.01–0.11; Passadore et al., 2017). Shallow, sheltered, inverse estuaries like Coffin Bay are highly productive (Kämpf, 2014); and reports of water quality indicate high nutrients loads particularly in the inner area of Coffin Bay (EPA, 2014). Moreover, Coffin Bay is an important nursery and feeding area for several fish and cephalopod species (DENR, 2010) that are known to constitute part of the diet of bottlenose dolphins in South Australia (Gibbs, Harcourt, & Kemper, 2011). Understanding the site fidelity and ranging patterns of dolphins within this area can contribute toward the development of spatial conservation measures of a significant dolphin population that is already immersed within a multiple‐use marine park, but for which there are no management plans.

In this study, we use photo‐ID data and genetic analyses of biopsy samples of southern Australian bottlenose dolphins collected between 2013 and 2015 in Coffin Bay to (i) determine individuals’ site fidelity patterns to the inner area, (ii) characterize ranging patterns of resident individuals, and (iii) assess sex differences in site fidelity and ranging patterns. Considering the apparent high productivity of Coffin Bay and the high density of dolphins inhabiting the inner area (Passadore et al., 2017), we predicted that dolphins would exhibit high degrees of site fidelity, range over relatively small areas, and males and females would show similar ranging patterns. Our results enhance our understanding of space use patterns in inshore dolphins and contribute to better informed decision making with regard to spatial management strategies aimed at protecting marine wildlife within marine parks in South Australia.

## 2 MATERIALS AND METHODS

### 2.1 Study area

**Figure 2:**
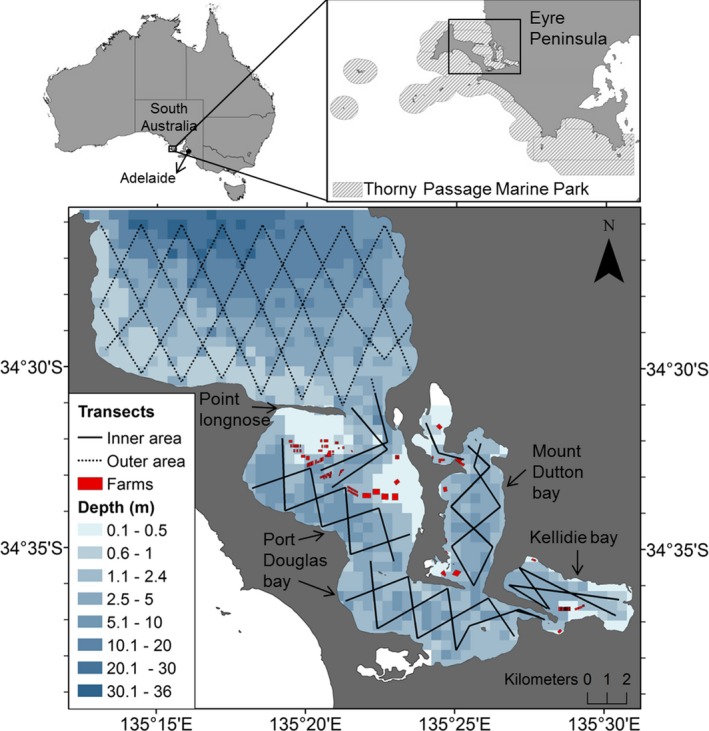
Map of the study area showing the location of Coffin Bay within the Thorny Passage Marine Park, Eyre Peninsula, South Australia. The zigzag transect layout (solid lines) used to cover the inner area (~123 km^2^) of Coffin Bay including Kellidie, Mount Dutton, and Port Douglas bays, and complementary transects (dashed lines) used to cover the outer area (~140 km^2^). The location of aquaculture oyster farms (Farms) and the bathymetry of the study area is shown (depth ranges are indicated by grid colors)

Coffin Bay is located within Thorny Passage Marine Park (TPMP), in the lower part of Eyre Peninsula, South Australia (Figure 2). It is divided into an inner (~123 km^2^) and an outer area (~140 km^2^) by a narrow and long (5 km) spit of land called Point Longnose, which restricts water exchange through a narrow opening between both areas. The inner area is a small inverse estuary that consists of several interconnected shallow (mean depth ~2.5 m) bays such as Port Douglas, Mount Dutton, and Kellidie (DEH, 2004; Saunders, 2009; Kämpf & Ellis, 2015; Figure 2). Evaporation rates exceeding precipitation between September and April lead to hypersaline conditions during austral summer; while in austral winter (June–August), the inverse pattern dilutes salinity leading to fresher waters mainly in Kellidie and Mount Dutton bays (Kämpf & Ellis, 2015). In most of this area, tides are of approx. 1.3 m (Saunders, 2009). Several types of habitats are found in the inner area including seagrass beds, subtidal sandflats, saltmarshes, salt creeks, low reefs, ponds, shallow pools, and limestone ledges (Saunders, 2009). The outer area extends from Point Longnose and connects the waters of the inner area with the Great Australian Bight. In the outer area, the depth increases from the shoreline to more than 25 m deep in the central and most exposed section of the bay, and its oceanographic conditions are influenced by several features of the Southern Ocean including upwelling events that occur off the continental shelf enhancing its productivity during the autumn months (DEH, 2004; Kämpf, Doubell, Griffin, Matthews, & Ward, 2004). In general, waters in the outer area have lower total nutrient loads than in the inner area; furthermore, water and habitat monitoring suggested that the inner area could be under stress from nutrient enrichment (EPA, 2014).

### 2.2 Survey design and data collection

Boat‐based surveys were conducted in Coffin Bay over six fieldwork seasons between September 2013 and October 2015 (Table 1). Surveys were carried out using a 6.5 m semirigid inflatable with twin 80 hp outboard motors or a 7.2 m rigid aluminum vessel with twin 115 hp outboard motors. Thorough coverage of the study area was obtained following two alternative “equal spaced zigzag” transect routes (Figure 2) designed with Distance 6.0 software (Thomas et al., 2010). Each route consisted of a total transect length of approximately ~55 km in the inner area and ~69 km in the outer area. The layout of transects maximized survey effort and ensured representative coverage of the different environmental conditions (e.g., depth, distance to shore, temperature, salinity, and habitat types) encountered within the study area. Shallow waters (<0.5 m in 20% of inner area), and the presence of oyster farms in the north‐east part of Port Douglas and south of Kellidie prevented access to these areas, thus boat surveys covered 85.5 km^2^ of the inner area and 140 km^2^ of the outer area (Figure 2). A total of 2–4 days were needed to complete a single survey of the entire inner area and 2–3 days to survey the entire outer area.

**Table 1:** Summary of the survey effort conducted in Coffin Bay, South Australia, between September 2013 and October 2015. Information for each fieldwork season is given, including period dates, the number of months surveyed, and the number of survey days on‐effort. Survey effort is also shown for inner and outer area separately including the total number of times each area was surveyed in its entirety (No. of surveys completed), the total kilometers of route surveyed (total survey effort), and the number of southern Australian bottlenose dolphin schools encountered on‐effort (no. of schools sighted)

Fieldwork season	Dates	No. of months surveyed	Days of survey on‐effort	Inner area			Outer area		
				No. of surveys completed	Total survey effort (km)	No. of schools sighted	No. of surveys completed	Total survey effort (km)	No. of schools sighted
1	September–November 2013	2.5	26	7	379.9	99	1	67	2
2	February–May 2014	3	29	8	435.6	113	3	208.8	8
3	July–September 2014	2	22	5	271	127	2	137.9	8
4	December 2014–January 2015	2	20	5	271	70	1	69	6
5	April–June 2015	2.5	27	7	382.4	144	2	137.9	6
6	August–October 2015	2	27	7	379.9	148	1	67	2
Total			151	39	2,119.8	701	10	687.6	32

Surveys were undertaken during daylight hours, at an average speed of 15 km/hr and under good weather conditions (i.e., Beaufort state ≤3, good–average visibility, no rain or fog, swell height ≤1 m). During surveys, three to five (mode = 4) observers searched for dolphins scanning at both sides of the boat, from −5° to 90° degrees of the transect, with 7 × 50 binoculars or with the naked eye. When a school of dolphins was sighted, the global positioning system (GPS) position at the transect was recorded, searching effort was suspended, and dolphins were approached slowly up to a distance of 10–20 m to record data on GPS position, school size, and composition (number of noncalves and calves) and to carry out photo‐ID and biopsy sampling. A school of dolphins was defined as all animals seen within a radius of 100 m (Wells, Irvine, & Scott, 1980) that were involved in similar (often the same) behavioral activities (modified from Connor, Mann, Tyack, & Whitehead, 1998). Distinguish among individuals’ age classes (adults, juveniles, and calves) in Coffin Bay is difficult as animals appear to be smaller in size in comparison with other study areas, thus individuals were categorized as noncalves (>1.5 m in length) and calves (≤1.5 m in length) as in Passadore et al. (2017). Only noncalves (i.e., adults and juveniles) were included in our analysis. Photographs of dorsal fins of individual dolphins were taken using a Nikon D300s DSLR camera with a 28–300 mm zoom lens and a Canon EOS 60D with a 100–400 mm zoom lens. Biopsy samples were obtained using a biopsy pole system for bow‐riding dolphins (Bilgmann, Griffiths, Allen, & Möller, 2007) or a PAXARMS remote biopsy system specifically designed for small cetaceans (Krützen et al., 2002). In the field, biopsy samples were preserved in a 20% dimethyl sulfoxide solution saturated with sodium chloride (Amos & Hoelzel, 1990), and after returning from field, they were frozen at −20°C until further analysis. We returned to the transect and resumed the survey effort once we obtained photographs of all or most of the individuals within a school, or when individuals were lost from sight for ≥10 min.

### 2.3 Data processing: photo‐ID and sexing

Dolphins were individually identified based on photographs of long‐lasting marks such as nicks, cuts, and deformities in the edges of their dorsal fins (Würsig & Jefferson, 1990; Würsig & Würsig, 1977). To minimize misidentification, all photographs taken were examined and given an overall quality score (“Q1” = “excellent”; “Q2” = “good;” and “Q3” = “poor”) based on the picture's focus, contrast, the angle of the dorsal fin to the camera, etc. Individual's dorsal fins were also classified into three distinctiveness categories (“D1” = “very distinctive,” “D2” = “average distinctive,” and “D3” = “Not distinctive”) according to the amount of information they presented (based on Urian et al., 2015; see full description of methodology in Passadore et al., 2017). The best images (right and/or left side) of each individual within a school were selected and were either matched with the already known individuals included in the Coffin Bay's fin catalog or incorporated into it with a new ID number. Only high‐quality photographs (i.e., Q1 and Q2) of distinctive individuals (i.e., D1 and D2) were included in the catalog and used for analyses. Information on date and location (GPS position) of the sighting was added to each individual's photograph cataloged. DISCOVERY (version 1.2.) was used to process, match, catalog, and manage all the photo‐ID data (Gailey & Karczmarski, 2013).

DNA from biopsy samples was extracted using a salting‐out protocol (Sunnucks & Hales, 1996), and fragments of the ZFX and SRY genes were amplified through the polymerase chain reaction (PCR) to determine the sex of sampled individuals (Gilson, Syvanen, Levine, & Banks, 1998). Individuals that were not biopsied, but were observed swimming accompanied by a dependent calf on ≥3 different survey days, were also considered adult females.

### 2.4 Data analysis

Given the high density of dolphins inhabiting the inner area (1.57–1.70 individuals/km^2^), their low temporary emigration rates (0.02; 95% CI: 0.01–0.11, Passadore et al., 2017), and the higher survey effort in the inner area compared to the outer area (Table 1; Figure S1), we focused our spatial analyses of site fidelity and ranging patterns on individuals identified in the inner area of Coffin Bay. We used data collected in the outer area to identify individuals whose space use expanded beyond the inner area during our study period and excluded them from the spatial analysis.

#### 2.4.1 Site fidelity

Three measures of site fidelity were estimated for each noncalf dolphin using information on date and location of photo‐identified animals: (i) site‐fidelity index, (ii) survey‐route sighting rate, and (iii) fieldwork‐season sighting rate. Site‐fidelity index for each individual was calculated as the ratio between the number of recaptures and the number of survey routes from its first capture to its last capture. An individual with a site‐fidelity index of zero indicates that it was captured only once during the study period, while an individual with a site‐fidelity index of one was captured in all survey routes after its first capture. The survey‐route sighting rate and fieldwork‐season sighting rate were calculated as the number of survey routes and fieldwork seasons a dolphin was identified as a proportion of the total number of survey routes and fieldwork seasons surveyed, respectively. In our study, survey‐route sighting rate ranged from 0.026 (individuals sighted in only one surveyed route) to one (individuals sighted in all 39 surveyed routes); while fieldwork‐season sighting rate ranged from 0.17 (individuals sighted in only one fieldwork season) to one (individuals sighted in all the six fieldwork seasons).

To identify clusters of individuals with similar degrees of site fidelity, the individuals’ values of site‐fidelity index, survey‐route sighting rate, and fieldwork‐season sighting rate were used in an agglomerative hierarchical clustering (AHC) analysis (Hunt et al., 2017; Zanardo et al., 2016). The AHC builds a dendrogram based on a bottom‐up clustering method, which starts with each observation as an individual cluster and successively combines the clusters according to their similarity until resulting into a single final cluster (Legendre & Legendre, 2012). The AHC analysis was built using Euclidean distance as the dissimilarity measure and Ward's method (minimum variance) as the agglomerative clustering algorithm since it is considered a robust approach (Singh, Hjorleifsson, & Stefansson, 2011; Ward, 1963). For each cluster in the dendrogram, the approximately unbiased (AU) probability values (i.e., *p‐*values) were obtained by generating 1,000 bootstrap resampling replications per cluster (Suzuki & Shimodaira, 2006). High AU *p*‐values indicate high confidence in the clusters and were used to define a cutoff point along the dendrogram (a dissimilarity threshold) to obtain the most suitable number of clusters (Singh et al., 2011). To test the overall validity of the clustering, the cophenetic correlation coefficient (CPCC) was also calculated. The CPCC measures the relation between the original dissimilarity matrix and the one (cophenetic matrix) obtained after the dissimilarities are recalculated by the clustering algorithm (Sokal & Rohlf, 1962). A high CPCC value (i.e., close to 1) indicates that the clustering is a good representation of the information contained in the original data (Bridge, 1993). All the clustering analysis was performed using the “pvclust” package (Suzuki & Shimodaira, 2006) in R version 3.2.3 (RCoreTeam, 2015).

To explore long‐term site fidelity to the inner area of Coffin Bay, we cross‐checked individuals identified during our study period (2013–2015) with 192 distinctive individuals which were identified during a pilot study between April and June of 2010 (Taylor, 2010). Taylor (2010) encountered a total of 153 dolphin groups during 16 boat‐based surveys which covered mainly the inner area of Coffin Bay and opportunistically the southern section of the outer area.

#### 2.4.2 Site fidelity toward specific areas

Individuals’ site fidelity toward specific areas within the inner area of Coffin Bay was explored by estimating the standard distance deviation (*S*_XY_) as in Parra et al. (2006). The *S*_XY_ represents the standard deviation of the distance of each point from their mean center and provides a good measure of the degree to which features are concentrated or dispersed around their mean center (Mitchel, 2005). The *S*_XY_ was calculated only for individuals that met all the following criteria: (i) were sighted in ≥7 different days during the study period; (ii) were classified as occasional or regular residents of the study area according to the AHC analysis; and (iii) were only observed in the inner area and never observed during the complementary surveys carried out in the outer area. The first criterion was established after determining that there was no significant relation (ANOVA, α ≤ .05) between the number of locations and the size of representative ranges estimated (see below) when using seven or more locations (ANOVA, *r*(110) = .160, *p *=* *.09). As the survey effort in the outer (i.e., complementary) area was lower than in inner area, the latter criteria aimed to reduce the likelihood of underestimating the area used by individuals that move beyond our main study area (inner area). As some individuals were sighted multiple times during the same day, we only included their first location of each day to avoid temporal autocorrelation in the analysis.

The *S*_XY_ was calculated as the standard deviation of the distance of each individual dolphin location to their mean center considering geographic coordinates in meters as follows (Mitchel, 2005):

$$S_{\mathrm{XY}}=\sqrt{\frac{\sum_{i=1}^{n}{\left(X_{i}-\overset{¯}{X}\right)}^{2}}{N}+\frac{\sum_{i=1}^{n}{\left(Y_{i}-\overset{¯}{Y}\right)}^{2}}{N}}$$

where *X*_*i*_ and *Y*_*i*_ are the geographic coordinates of the *i* location of an individual, $\overset{¯}{X}$ and $\overset{¯}{Y}$ are the coordinates of the mean center of all the locations of that individual, and *N* is the number of locations for that individual dolphin. Low values of *S*_XY_ indicate that the locations of an individual are limited to a small area and thus has high site fidelity for a particular area within Coffin Bay. The *S*_XY_ of each individual was calculated using the spatial statistics tools of ArcGIS 10.3.1, using the Universal Transverse Mercator (UTM) Zone 35° South projection and based on the WGS 1984 datum. Difference in *S*_XY_ between sexes was evaluated in R version 3.2.3 (RCoreTeam, 2015) with a Kruskal–Wallis test at α ≤ .05.

#### 2.4.3 Ranging patterns

Ranging patterns were estimated for all individuals that followed the same criteria mentioned above for *S*_XY_ analysis. To determine the size of the area used by each individual (i.e., representative range) within inner Coffin Bay, we used the kernel method, which estimates a probability density function that represents the utilization distribution (UD) of an individual (Silverman, 1986; Van Winkle, 1975; Worton, 1989). As the coastline separating the system of bays and channels of Coffin Bay impose physical barriers to dolphins’ movements, we used the “kernel interpolation with barriers tool” available from the Geostatistical Analyst Toolbox in ArcGIS 10.3.1. This tool uses the shortest distance between points without intersecting the barrier (Gribov & Krivoruchko, 2011), which allows to obtain accurate estimates of the dolphins’ representative ranges (i.e., 95% kernel range, Worton, 1995) area without biases imposed by the coastline (e.g., Sprogis et al., 2016; Wells et al., 2017).

The settings of the kernel interpolation with barriers analysis were kept consistent between individuals to ensure comparable results among individuals. The output grid cell size was set to 200 × 200 m, which allowed sufficient information to be included in the narrow channels and bay entrances of the study area. A first‐order polynomial was selected as the kernel function, and the default value of 50 was used for the ridge parameter. The bandwidth value (i.e., search radius that determines which surrounding location points will contribute to the kernel density) was chosen by visual inspection (Wand & Jones, 1995) after running several trials with different bandwidth values (bandwidth range = 500–6,000; Figure S2). If the bandwidth is too small, it can generate a fragmented UD with various components and result in negatively biased home range estimates; if the bandwidth is too large, the UD can be excessively smooth and the home range is overestimated (Gitzen, Millspaugh, & Kernohan, 2006; Kie et al., 2010). After visual inspection of the different trials, the bandwidth selected for the analysis was fixed at 3,000 m because the UDs obtained showed little fragmentation and were not overly smooth. The bandwidth was held constant across the plane for a fixed kernel.

Differences in representative ranges between sexes were evaluated using a Kruskal–Wallis test as for the *S*_XY_. Finally, to explore individuals’ space use over the long term, we plotted the location of individuals cataloged in 2010 (Taylor, 2010) and checked if they fell within the representative ranges estimated in this study.

## 3 RESULTS

We completed 39 survey routes of the inner area of Coffin Bay between September 2013 and October 2015 (Table 1), covering ~2,120 km of transect on effort. A total of 701 schools of dolphins were encountered (Table 1), and 179 distinctive noncalf individuals were photo‐identified. We were able to determine the sex of 64% (*n* = 114) of the photo‐identified dolphins (62 females and 52 males, Table 2) based on genetic analysis of 103 biopsy samples and the observation of presumed mother–calf associations for 11 individuals. The sex ratio of biopsied individuals was balanced, with 1.02 males per one female.

**Table 2:** Site‐fidelity measures of southern Australian bottlenose dolphins in inner Coffin Bay including site‐fidelity index, survey‐route sighting rate, and fieldwork‐season sighting rate. The mean and standard deviation (Mean ± *SD*), lower and upper 95% confidence intervals (CI 95%), and minimum and maximum (Min–Max) values are shown for all dolphins photo‐identified and by sex (females, males, and unknown sex)

	Total	Female	Male	Unknown
*N*	179	62	52	65
Site‐fidelity index				
Mean ± *SD*	0.30 ± 0.16	0.34 ± 0.14	0.34 ± 0.15	0.22 ± 0.16
CI 95%	0.28–0.34	0.29–0.35	0.32–0.38	0.19–0.29
Min–Max	0–0.67	0.04–0.65	0.14–0.60	0–0.59
Survey‐route sighting rate				
Mean ± *SD*	0.28 ± 0.15	0.32 ± 0.13	0.33 ± 0.14	0.22 ± 0.14
CI 95%	0.26–0.30	0.27–0.35	0.30–0.36	0.16–0.26
Min–Max	0.03–0.64	0.03–0.64	0.03–0.64	0.03–0.54
Fieldwork‐season sighting rate				
Mean ± *SD*	0.78 ± 0.27	0.85 ± 0.21	0.85 ± 0.25	0.67 ± 0.31
CI 95%	0.79–0.87	0.88–0.95	0.96–1.04	0.70–0.96
Min–Max	0.17–1	0.67–1	0.67–1	0.17–1

During the complementary surveys (10 survey routes, ~688 km of transect effort) of the outer area, a total of 32 schools of dolphins were encountered (Table 1) and 96 noncalves dolphins photo‐identified. Half of the photo‐identified individuals in the outer area (*n* = 48) were also observed in the inner area, so they were excluded from the *S*_XY_ and representative ranges analysis. A total of 131 individuals (58% of the individuals photo‐identified in the entire Coffin Bay) were found exclusively in the inner area.

### 3.1 Site fidelity

Out of the 179 noncalves individuals photo‐identified in the inner area, fifteen were seen only once. The remaining 164 individuals were sighted between two and 25 survey routes in the inner area. Measures (mean ± *SD*) of site fidelity for all photo‐identified individuals in the inner area were moderately high (site‐fidelity index = 0.30 ± 0.16, survey‐route sighting rate = 0.28 ± 0.15, and fieldwork‐season sighting rates = 0.78 ± 0.27), indicating a large proportion of the individuals were sighted regularly in this area (Table 2). Individuals were seen on average during 11 (*SD* = 5.7) of the 39 survey routes. Forty‐six percent of photo‐identified dolphins (*n* = 82) were seen in all fieldwork seasons surveyed, and 71% over all 3 years sampled. Values of the three site‐fidelity measures were also high and similar between females and males, indicating both sexes used the area regularly over the study period (Table 2).

**Figure 3:**
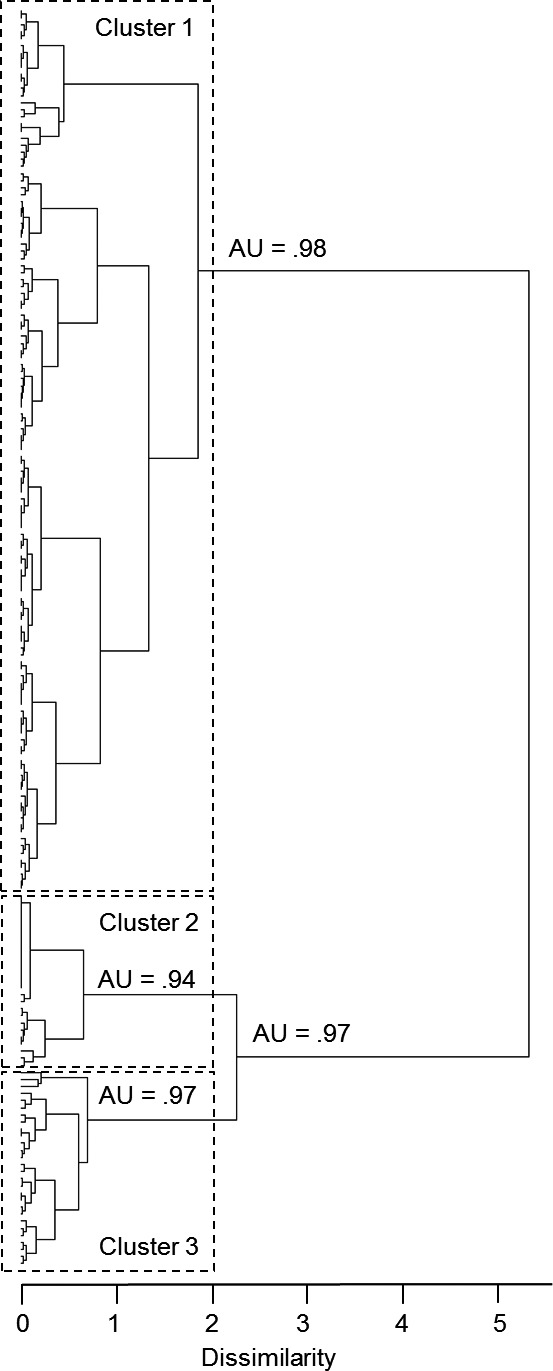
Agglomerative hierarchical clustering (AHC) dendrogram of southern Australian bottlenose dolphins in inner Coffin Bay obtained based on three measures of individuals’ site fidelity: site‐fidelity indices, survey‐route sighting rate, and fieldwork‐season sighting rate. Rectangles indicate three clusters (dissimilarity threshold = 2.0): Cluster 1 (“regular residents”), Cluster 2 (“occasional residents”), and Cluster 3 (“occasional visitors”). The approximately unbiased (AU) probability values of these three clusters are shown on the dendrogram

Three main clusters of individuals were identified from the AHC analysis (dissimilarity threshold = 2.0) based on site‐fidelity measures (Figure 3; Table 3). The high value of the cophenetic correlation coefficient (CPCC = 0.77) and approximately unbiased *p*‐values (AU *p*‐values = .94–.98) indicated that the dissimilarities among observations were well represented by the clusters in the dendrogram. Cluster 1 consisted of 125 individuals with relatively even numbers of males (*n* = 42) and females (*n* = 48), and the highest values of site‐fidelity indices, and survey‐route and fieldwork‐season sighting rates (Table 3). These individuals were sighted on average over 13 survey routes and on five or all six fieldwork seasons; thus, we consider them as “regular residents” of the inner area of Coffin Bay. Cluster 2 comprised 28 individuals (five males and 10 females) sighted in the inner area over seven survey routes on average, and in at least three fieldwork seasons, these dolphins were considered “occasional residents” to the inner area. Cluster 3 consisted of 26 individuals (five males and four females) sighted from one to five times, and in no more than two fieldwork seasons, these were considered “occasional visitors” to the inner area (Table 3).

**Table 3:** Site‐fidelity indices, and survey‐route and fieldwork‐season sighting rates for the three clusters of southern Australian bottlenose dolphins identified in inner Coffin Bay using the agglomerative hierarchical clustering (AHC) analysis. Mean and standard deviation (±*SD*), lower and upper 95% confidence intervals (CI 95%), and minimum and maximum (Min–Max) values are shown for all dolphins photo‐identified and by sex (females, males, and unknown sex) per cluster

	Cluster 1				Cluster 2				Cluster 3			
	Total	Females	Males	Unknown	Total	Females	Males	Unknown	Total	Females	Males	Unknown
*N*	125	48	42	35	28	10	5	13	26	4	5	17
Site‐fidelity index												
Mean ± *SD*	0.36 ± 0.11	0.37 ± 0.12	0.39 ± 0.11	0.33 ± 0.11	0.23 ± 0.11	0.26 ± 0.11	0.23 ± 0.05	0.2 ± 0.12	0.03 ± 0.06	0.09 ± 0.12	0.04 ± 0.06	0.02 ± 0.03
CI 95%	0.33 to 0.37	0.31 to 0.37	0.35 to 0.41	0.28 to 0.36	0.15 to 0.22	0.19 to 0.33	0.21 to 0.25	0.15 to 0.19	−0.02 to 0.02	−0.06 to 0.14	−0.04 to 0.04	−0.02 to 0.02
Min–Max	0.13 to 0.67	0.18 ± 0.67	0.2 ± 0.67	0.13 ± 0.59	0.08 to 0.55	0.11 ± 0.44	0.15 ± 0.29	0.08 ± 0.55	0 to 0.23	0 ± 0.23	0 ± 0.14	0 ± 0.12
Survey‐route sighting rate												
Mean ± *SD*	0.35 ± 0.1	0.36 ± 0.11	0.38 ± 0.1	0.32 ± 0.1	0.18 ± 0.06	0.2 ± 0.06	0.19 ± 0.06	0.17 ± 0.06	0.05 ± 0.03	0.05 ± 0.04	0.05 ± 0.03	0.04 ± 0.03
CI 95%	0.31 to 0.35	0.3 to 0.36	0.34 to 0.4	0.28 to 0.34	0.16 to 0.2	0.16 to 0.26	0.11 to 0.19	0.13 to 0.17	0.02 to 0.04	0 to 0.08	0.02 to 0.04	0.02 to 0.04
Min–Max	0.13 to 0.64	0.18 ± 0.64	0.21 ± 0.64	0.13 ± 0.54	0.1 to 0.33	0.1 ± 0.28	0.15 ± 0.28	0.1 ± 0.33	0.03 to 0.13	0.03 ± 0.1	0.03 ± 0.1	0.03 ± 0.13
Fieldwork‐season sighting rate												
Mean ± *SD*	0.94 ± 0.08	0.94 ± 0.08	0.96 ± 0.07	0.93 ± 0.08	0.6 ± 0.08	0.63 ± 0.07	0.57 ± 0.09	0.58 ± 0.09	0.22 ± 0.08	0.25 ± 0.1	0.2 ± 0.07	0.23 ± 0.08
CI 95%	0.98 to 1.02	0.96 to 1.04	0.96 to 1.04	0.95 to 1.05	0.62 to 0.72	0.67 to 0.67	0.38 to 0.62	0.43 to 0.57	0.12 to 0.22	0.12 to 0.38	0.17 to 0.17	0.11 to 0.23
Min–Max	0.83 to 1	0.83 ± 1	0.83 ± 1	0.83 ± 1	0.5 to 0.67	0.5 ± 0.67	0.5 ± 0.67	0.5 ± 0.67	0.17 to 0.33	0.17 ± 0.33	0.17 ± 0.33	0.17 ± 0.33

The cross‐checking of catalogs showed that at least 67% (*n* = 119) of the individuals photo‐identified during 2013–2015 were previously cataloged in the pilot study of 2010 (Taylor, 2010). These 119 individuals corresponded to 75% of dolphins considered members of cluster 1, 50% of cluster 2, and 42% of cluster 3. This suggests that dolphins of all clusters, including the ones considered occasional visitors, exhibit long‐term site fidelity to the study area.

### 3.2 Site fidelity toward specific areas

Out of the 131 noncalves individuals photo‐identified exclusively in the inner area, 112 (45 females, 36 males and 31 dolphins of unknown sex) were recorded at least seven times, including 99 that were classified as “regular residents” and 12 as “occasional residents” by the AHC. This dataset was used for analysis of standard distance deviation (*S*_XY_) and ranging patterns. For dolphins included in the spatial analysis, the mean (±*SD*) number of locations and the time interval between locations per individual was 14.3 ± 4.3 (median = 14) and 56 ± 17 days (median = 52), respectively.

**Figure 4:**
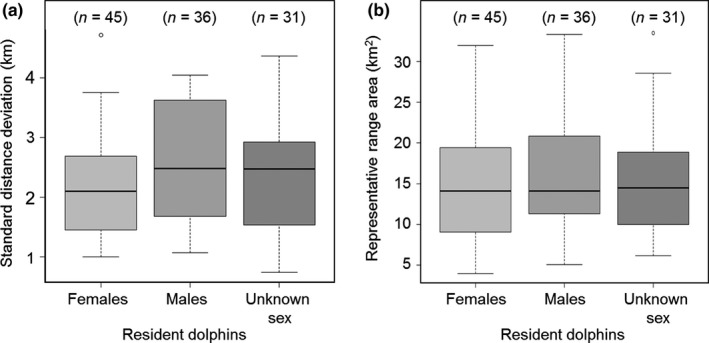
Box plots of (a) standard distance deviation and (b) representative range area for females (*n* = 45), males (*n* = 36), and individuals of unknown sex (*n* = 31) of southern Australian bottlenose dolphins residents to the inner area of Coffin Bay. The bold line indicates the median value, the rectangle spans from the first quartile to the third quartile, and the whiskers above and below the box show the locations of the minimum and maximum values, respectively. Circles beyond the maximum value represent the outliers

The *S*_XY_ of individuals ranged from 0.7 to 4.7 km (Figure 4a), with a mean (±*SD*) of 2.3 ± 0.9 km (median = 2.3 km), suggesting that dolphins had strong site fidelity to specific and relatively small areas within the inner area of Coffin Bay. The mean (±*SD*) *S*_XY_ for females (2.2 ± 0.8 km), males (2.5 ± 1.0 km), and for individuals of unknown sex (2.4 ± 0.9 km; Figure 4a) was similar, with no significant differences (Kruskal–Wallis, χ^2^ = 3.807, *df* = 2, *p *=* *.149).

### 3.3 Ranging patterns

Overall, representative ranges were small and restricted to particular areas. The area of an individuals’ representative range (95% kernel range) varied from 3.9 to 33.5 km^2^, with a mean (±*SD*) of 15.2 ± 6.8 km^2^ (median = 14.1). The size of the representative range for females (14.7 ± 7.0 km^2^), males (15.6 ± 6.6 km^2^), and for individuals of unknown sex (15.4 ± 7.0 km^2^; Figure 4b) was similar and showed no significant differences (Kruskal–Wallis, χ^2^ = 0.426, *df* = 2, *p *=* *.808). The majority of females (56%) and males (55%) had representative ranges smaller than 15 km^2^, with only a few individuals (9% females and 8% males) using areas larger than 25 km^2^.

**Figure 5:**
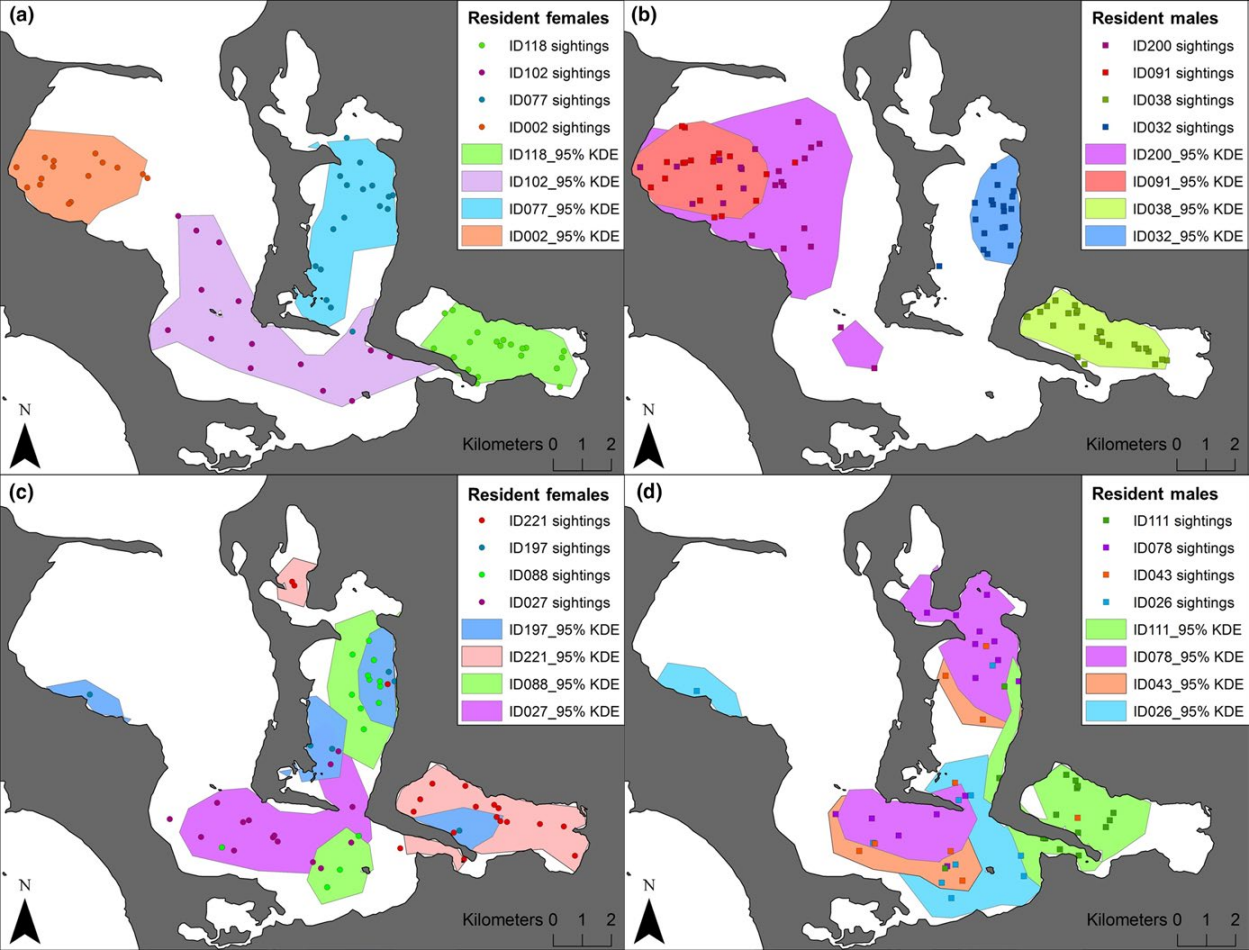
Examples of the representative ranges (95% kernel) of males and female southern Australian bottlenose dolphins encountered within the inner area of Coffin Bay between September 2013 and October 2015. Four (a) females and (b) males with representative ranges restricted to particular bays, and (c) females and (d) males with representative ranges covering multiple bays

The representative range of 56% of the individuals (63 of 112) was restricted to a particular bay within the inner area of Coffin Bay (see examples in Figure 5a,b; Figure S3). The other 44% of individuals’ representative ranges covered multiple areas within Coffin Bay (see examples in Figure 5c,d; Figure S3).

Out of the 112 resident individuals included in the spatial analysis, 78 (70%) were previously photo‐identified during the 2010 pilot study (Taylor, 2010). Furthermore, records of 2010 indicated that the sightings of 62 of them fell within the representative ranges estimated in the 2013–2015 study period; while nine individuals were observed in 2010 at less than 1 km distance from their current representative range, and the remaining seven dolphins were seen at further distances.

## 4 DISCUSSION

Marine mammal site fidelity and ranging patterns can provide important information about the space use patterns and relative significance of particular areas to individuals, groups, and populations which are relevant for delineating conservation and management strategies for at‐risk species. This study shows that the majority of southern Australian bottlenose dolphins inhabiting the inner area of Coffin Bay, South Australia, exhibit a high degree of site fidelity, with both sexes ranging over relatively small areas. Furthermore, a large proportion (56%) of individuals within the inner area appears to restrict their space use to particular embayments. High levels of site fidelity and restricted ranging patterns in dolphins are hypothesized to occur in areas where resources are spatially and temporally predictable (Gowans et al., 2008). The site fidelity and range characteristics of bottlenose dolphins reported here are concordant with theoretical models of site fidelity (Gowans et al., 2008; Switzer, 1993) and our predictions based on the apparent high biological productivity of the area, the absence of sex‐biases in demographic parameters, and the high‐density population inhabiting the inner Coffin Bay area (Passadore et al., 2017). These findings emphasize the importance of habitat quality as a major factor driving site fidelity and movement patterns in highly mobile marine mammals and highlight the conservation value of the inner area of Coffin Bay for southern Australian bottlenose dolphins.

When comparing home range studies, caution must be taken because different methodologies (e.g., minimum convex polygon, adaptive or fixed Kernel) can produce different estimates of ranging patterns (de Faria Oshima & de Oliveira Santos, 2016). Taking this into account, we found that the sizes of the representative ranges of resident southern Australian bottlenose dolphins in the inner area of Coffin Bay seem to be smaller than mean sizes reported for inshore bottlenose dolphin species elsewhere (see comparable examples in Table S1). However, the size of the representative ranges observed in our study was within the ranges reported for other inshore delphinids inhabiting small bays (e.g., 15.22 km² for *Sotalia flluviatilis* in Baía Norte, Santa Catarina, Brazil, Flores & Bazzalo, 2004; 13.5 km^2^ for *Sotalia guianensis* in Cananéia estuary, São Paulo, Brazil, de Faria Oshima & de Oliveira Santos, 2016). These bays share characteristics with Coffin Bay that may be promoting such spatial patterns; they all are shallow systems (mean depth <7 m), located within (or part of) marine protected areas, and are productive systems considered nursery areas of several fish species that are part of the dolphins diet (Flores & Bazzalo, 2004; de Faria Oshima & de Oliveira Santos, 2016; see below further references for this study). Broad‐scale models of home range in mammals have shown that body size and sex are important predictor of home range size and that (i) marine mammals tend to range over larger areas than terrestrial mammals of similar size (Tucker et al., 2014) and (ii) adult males tend to have larger ranges than adult females. At finer‐scales, however, there is great variability in space use patterns within and among species even when they share similar characteristics (e.g., similar body size and diet, and inhabit similar environments) (Table S1). Such intra‐and interspecific differences in space use among bottlenose dolphins are likely driven by a combination of multiple factors acting at finer‐scales rather than body size and sex alone.

The degree of site fidelity an individual has to a particular location, and its ranging patterns is a reflection of extrinsic factors such as environmental conditions, habitat quality, distribution of food resources, potential mating partners and predators, intra‐ and interspecific competition, and population density as well as intrinsic components, such as body size, individual's experience, sex, and age (Duncan, Nilsen, Linnell, & Pettorelli, 2015; McLoughlin & Ferguson, 2000; Saïd et al., 2009; Switzer, 1993, 1997). Simulations and empirical studies across different mammal species have shown that, among these factors, food availability and population density play a pervasive role in determining the size, shape, and location of home ranges, with animals distributing themselves in a way that maximizes the use of spatially distributed resources while minimizing competition with conspecifics (Duncan et al., 2015; Mitchell & Powell, 2012; Šálek, Drahníková, & Tkadlec, 2015; Schoepf, Schmohl, König, Pillay, & Schradin, 2015). In general, these studies show that home range size decreases with (i) increasing food availability, because individuals can access food more easily and thus save energy, and (ii) increasing population density, because individuals space use patterns are constrained by competitive interactions with neighboring individuals. A high density of dolphins is found in the inner area of Coffin Bay waters (1.57–1.70 individuals/km^2^, Passadore et al., 2017), with resident dolphins remaining close (<5 km) to their mean center of use and showing restricted representative ranges (<35 km^2^). Studies on bottlenose dolphins have shown that some populations have low fidelity and use large areas (e.g., *Tursiops truncatus*, Ballance, 1992; Defran, Weller, Kelly, & Espinosa, 1999), while others have strong site fidelity and small ranging patterns (*Tursiops aduncus*, Sprogis et al., 2016; *T. truncatus*, Gubbins, 2002; Ingram & Rogan, 2002; Urian et al., 2009; Brusa, Young, & Swanson, 2016; Wells et al., 2017). The latter usually occurs when dolphins inhabit sheltered and highly productive waters, such as estuaries. For example, in Bunbury, Western Australia, bottlenose dolphins (*T. aduncus*) which were more often sighted in productive sheltered habitats (i.e., bay, estuary, and riverine waters) had smaller representative ranges than dolphins that predominately use less productive open waters (Sprogis et al., 2016). In areas with a surplus of food, increases in population density can lead to an increase in home range overlap between individuals and sharing of food resources, which can lead to intraspecific competition for food (Schoepf et al., 2015). Small and nonoverlapping ranging patterns among individuals within a population may constitute a strategy to avoid competition for food resources in an area highly populated by cospecifics (Gowans et al., 2008; Mcloughlin, Ferguson, & Messier, 2000; Schoepf et al., 2015; Schradin et al., 2010). Our results support the hypothesis that the apparent high productivity of the inner area of Coffin Bay likely provides enough resources for dolphins, allowing for optimal foraging efficiency within small representative ranges. Furthermore, the high density of dolphins found in the inner area of Coffin Bay, and the potential intraspecific competition associated with it, might also contribute to the small ranges and spatial segregation observed among a large proportion of the resident individuals.

When dolphins have high site fidelity to an area and restricted ranging patterns, they will likely become familiar with the quality of habitats and the predictability of resources and develop social bonds with other individuals using the same area (Connor & Krützen, 2015; Connor et al., 2000; Lusseau et al., 2003; Urian et al., 2009). Familiarity with resources and conspecifics together with long‐lasting social bonds allows for information transfer among members of a community on the distribution of food resources and predators, contributing to maximize individuals’ fitness and survival (Lusseau et al., 2003; Switzer, 1993, 1997). The high site fidelity of dolphins occurring in the inner area of Coffin Bay is likely favored by a lower predation risk compared to the outer area and coastal waters of South Australia. The inner area is characterized by shallow waters and a narrow connection with the outer area, which may restrict the access of predators to the study area. One of the main predators of dolphins in coastal waters of South Australia is the white shark (*Carchharodon carcharias*), which can occur close to shore although they seem to prefer waters of <100 m depth (Bruce, Stevens, & Malcolm, 2006). Additionally, the high diversity of habitats (Miller, Westphalen, Jolley, & Brayford, 2009) and differences in environmental conditions found in Coffin Bay (Kämpf & Ellis, 2015) likely result in different fish assemblages across its different embayments. A contemporary study performed in autumn and spring 2015 revealed that, in fact, fish assemblage composition differ among embayments of the inner area (i.e., Kellidie vs. Mount Dutton vs. north of Port Douglas vs. south of Port Douglas) (S. Whitmarsh, personal communication, 14 March 2017). Consequently, individuals inhabiting each embayment may have developed different feeding habits in response to variation in habitat and associated prey resources. Such potential feeding differences and spatial segregation may also be strengthened by social structure patterns. The population of bottlenose dolphins inhabiting the inner area of Coffin Bay is socially structured, with at least two well‐differentiated communities occurring in different embayments, one in Port Douglas and the other in Kellidie‐Mount Dutton bays (Diaz‐Aguirre, 2017). Further studies integrating predation risk, social structure, and feeding ecology should improve our understanding of the extrinsic drivers of the high residency and fine‐scale spatial structure observed for this highly mobile species in such a small area and whether such patterns offer fitness improvements.

Determining the factors that shape site fidelity and ranging patterns of highly mobile marine species that spend most of their time underwater such as dolphins, represents a challenging field of research. Ranging patterns of dolphins have been studied using radio‐tracking (Martin & Silva, 2004; Owen, Wells, & Hofmann, 2002), satellite‐tracking (Wells et al., 1999, 2017), and photo‐ID techniques (Owen et al., 2002; Sprogis et al., 2016) as we used here. Although radio, and especially satellite‐tracking approaches, can provide very detailed information on animal movement and ranging patterns, usually only a few individuals from a population can be studied, resulting in ranging patterns that may not be representative of the entire population (Castro et al., 2014; Irvine et al., 2014). Photo‐ID is a noninvasive mark–recapture technique that has been applied to study the fidelity and space use patterns of several species, including highly mobile marine animals such as sharks (Brooks, Rowat, Pierce, Jouannet, & Vely, 2010; Domeier & Nasby‐Lucas, 2007), whales (Craig & Herman, 1997; Dorsey, Stern, Hoelzel, & Jacobsen, 1990), and dolphins (de Faria Oshima & de Oliveira Santos, 2016; Gubbins, 2002; Sprogis et al., 2016). However, one of the limitations of using photo‐ID to estimate the site fidelity and ranging patterns of highly mobile species is that it can only be conducted during daylight hours in good weather conditions and is limited to the study area and period covered by researchers. Nonetheless, a study comparing home ranges of bottlenose dolphins determined using mark–recapture data from photo‐ID surveys vs. radio‐tracking data showed that both approaches produced very similar patterns for individuals that appeared to be year‐round residents to the surveyed area (Owen et al., 2002). We acknowledge that this study carries the limitations imposed by photo‐ID; our data were collected only during daytime, with some time gaps (i.e., 2–3 months) between fieldwork seasons and over a short period of time (2 years) relative to the dolphins’ normal life‐span (*ca*. 40 years). However, our previous study at the population level indicated that emigration rates from the inner area are very low (Passadore et al., 2017), and cross‐checking of individuals identified during our study period (2013–2015) with individuals identified in 2010 (Taylor, 2010) indicated long‐term site fidelity to the inner area. Furthermore, we limit our analysis to resident individuals based on their sighting patterns across the study period. Thus, we consider that our approach provides robust estimates of the space use of the resident dolphins within the inner area of Coffin Bay and a solid platform for future investigations into their site fidelity and ranging patterns.

### 4.1 Implications for conservation

Marine mammal populations exhibiting high levels of site fidelity and restricted ranging patterns are particularly susceptible to localized anthropogenic pressures such as habitat degradation and loss, entanglements in marine debris, interaction with fisheries (i.e., bycatch or reduction in prey availability due to overfishing), pollution, among others (Atkins et al., 2016; Currey, Dawson, & Slooten, 2007; Monk, Charlton‐Robb, Buddhadasa, & Thompson, 2014; Rojas‐Bracho, Reeves, & Jaramillo‐Legorreta, 2006). At the same time, such populations have the potential of being protected using area‐based management measures, especially if specific strategies are established and enforced to reduce the local threats (Augé, Chilvers, Moore, & Davis, 2014; Gormley et al., 2012). Although marine mammals are considered species of ecological value within the management plan for the TPMP in which Coffin Bay is located (Bryars et al., 2016), there are no strategies directed toward the protection of dolphins. The zoning in most of the TPMP, including Coffin Bay waters, allows human activities (e.g., oyster aquaculture, recreational fishing, water sports, and tourism cruises, Saunders, 2009; DENR, 2010) that could be negatively impacting upon the dolphins. Due to their high site fidelity and restricted ranging patterns, it is likely that resident individuals inhabiting specific areas may be facing different threats. For instance, Mount Dutton and Kellidie bays are particularly vulnerable to harmful algae blooms and pollution because of their relatively slow flushing (water age of ~3 months; Kämpf & Ellis, 2015), which can result in cascade effects producing mortalities of prey (e.g., PIRSA, 2014) and potentially also affecting dolphins. The spatial distributions of threats to southern Australian bottlenose dolphins, however, are poorly understood. Therefore, future research is needed to map the distribution of major threats to dolphins in the area. This, together with the results presented here, should be considered in the zoning arrangements and management strategies of TPMP plan, which is scheduled to be reviewed in 2022.

## CONFLICT OF INTEREST

None declared.

## AUTHOR CONTRIBUTIONS

Cecilia Passadore (CP), Guido J. Parra (GJP), and Luciana Möller (LM) conceived and designed the study. CP and Fernando Diaz‐Aguirre (FDA) collected the data. CP analyzed the data with advice and contributions to data analysis from GJP, LM, and FDA. FDA processed the genetic samples. CP wrote the manuscript with contributions to drafting, critical review, and editorial input from GJP, LM, and FDA.
