# Supplementary material for: High site fidelity and restricted ranging patterns in southern Australian bottlenose dolphins
Source: Ecol Evol. 2017 Nov 26;8(1):242–56. doi: 10.1002/ece3.3674 (PMC5756869; doi:10.1002/ece3.3674)
Supplement: Supplementary file 1 [file ECE3-8-242-s001.pdf]

High site fidelity and restricted ranging patterns in southern Australian bottlenose dolphins

Cecilia Passadore[1,*], Luciana Möller[1,2], Fernando Diaz-Aguirre[1,2], Guido J. Parra[1].

[1]Cetacean Ecology, Behaviour and Evolution Lab, School of Biological Sciences, Flinders University, South Australia.

[2]Molecular Ecology Lab, School of Biological Sciences, Flinders University, South Australia.

[*]Corresponding author

Contact details:

Corresponding author: Cecilia Passadore

Email: cecipass8@gmail.com

Address: Cetacean Ecology, Behaviour and Evolution Lab (CEBEL); School of Biological Sciences; Faculty of Science and Engineering; Flinders University. Sturt Road, Bedford Park, South Australia, 5042, Australia.

Phone: +61 8 82013865

Supplementary material

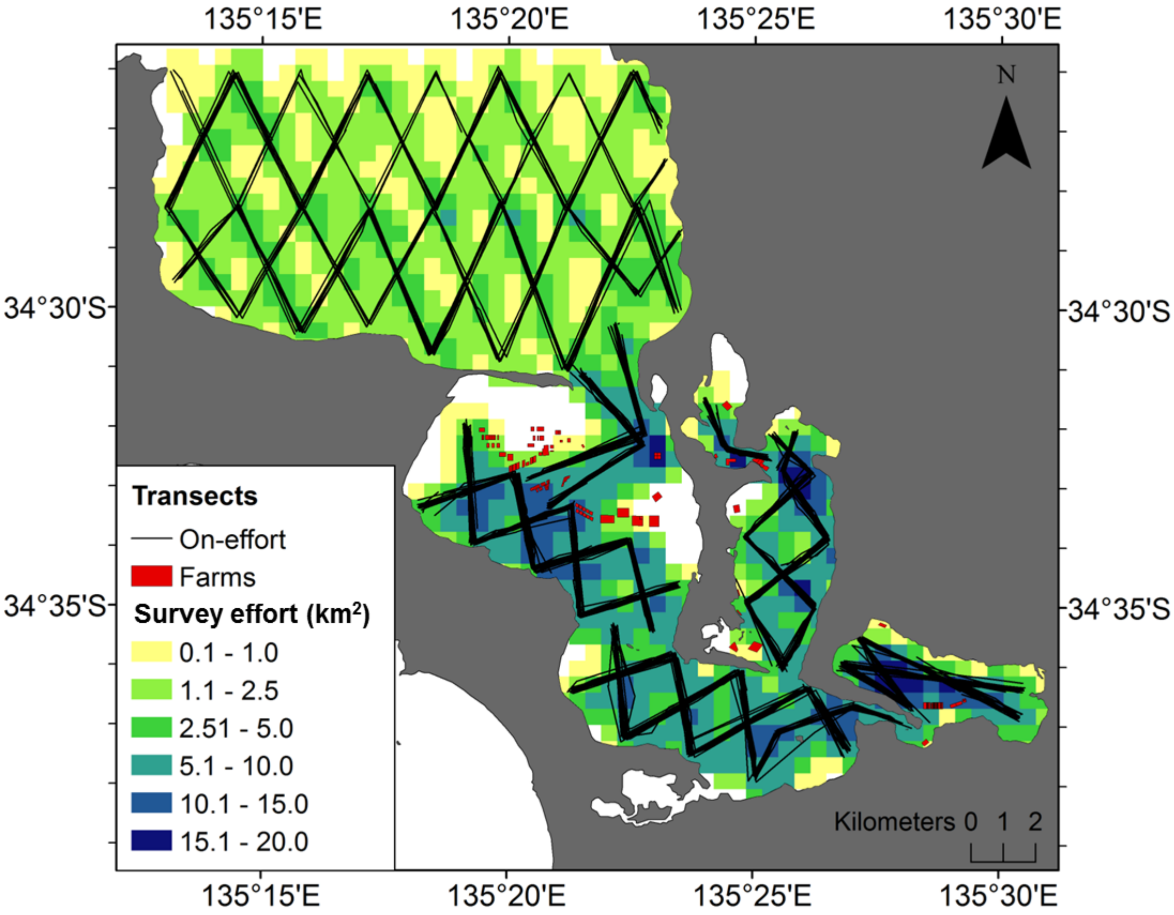

Fig. S1. Survey effort done in Coffin Bay between September 2013 and October 2015. The layout of the transects surveyed on effort (lines) is shown. The coloured 500 x 500 m grid cells represent the survey effort in km^2 determined considering an area buffer of 500 m to the sides of each transect surveyed.

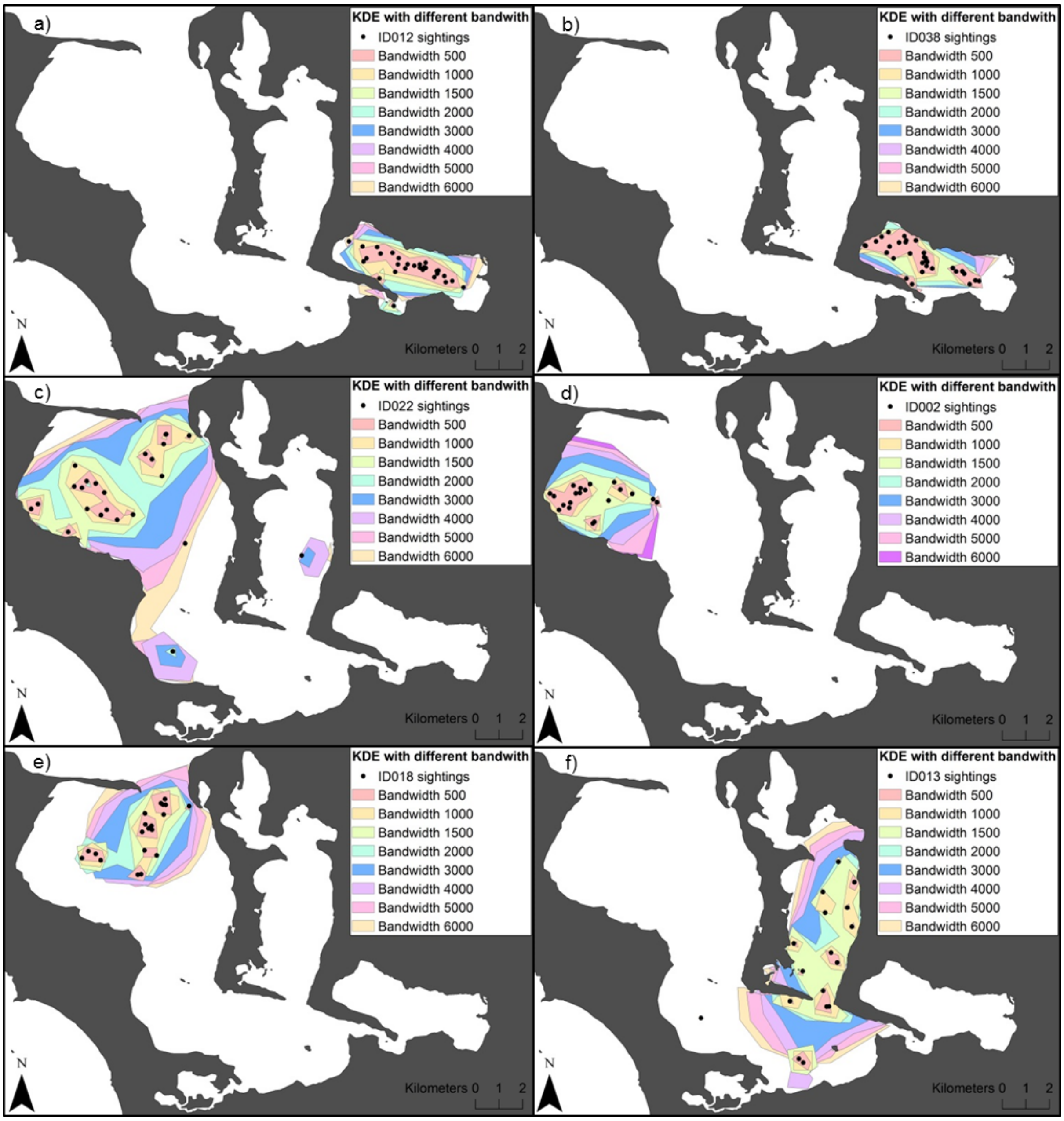

Fig. S2. Examples of Kernel density estimates for six individual dolphins that represented the ranges that were observed of the individuals in the field (a) male ID012; b) male ID038; c) female ID022; d) female ID002; e) female ID018; and f) male ID013) using consistent settings of grid cell size (i.e. 200 x 200 m), kernel function (i.e. first order polynomial), and ridge parameter (i.e. 50), while using different trials of bandwidth value at: 500; 1,000; 1,500; 2,000; 3,000; 4,000; 5,000; and 6,000 m. After visual inspection of the different trials, the bandwidth selected for the analysis was fixed at 3,000 m because the UDs obtained with this value were not fragmented and not overly smooth.

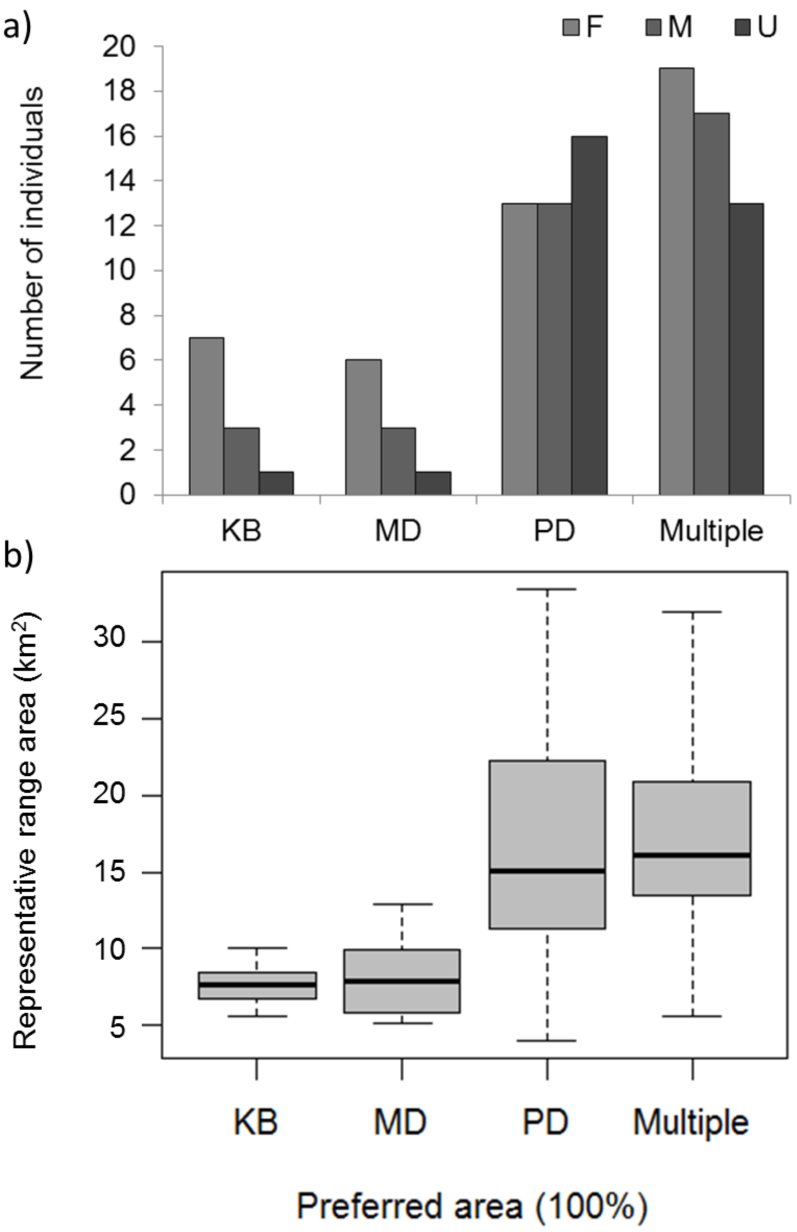

Fig. S3. Southern Australian bottlenose dolphins photo-identified between September 2013 and October 2015 in just one of the bays of the inner area of Coffin Bay (KB, Kellidie Bay; MD, Mount Dutton Bay; and PD, Port Douglas Bay) and in 'Multiple' bays. a) Distribution of frequency of individuals (F, females; M, males; and U, unknown sex) per bay. b) Boxplot of the size of representative ranges for individuals of each bay. In boxplots, the bold line indicates the median value, the rectangle spans from the first quartile to the third quartile, and the whiskers above and below the box show the locations of the minimum and maximum values, respectively.

Table S1. Representative range (95% kernel range) sizes for different delphinid populations and species. The species, study area, habitat type, period of study and duration, age class of individuals, size of individuals' representative range (mean ± SD), methodology and references for each study are shown. For comparative purposes, only examples using kernel methods to estimate representative ranges are presented here. Ranging patterns are shown for all individuals studied and separated by sex if available.

Species	Study area	Habitat type	Study period (duration)	Age class	Size of individuals' representative range (km^2) (Mean ± SD)	Methods	Reference
Tursiops cf. *australis*	Inner area of Coffin Bay, South Australia, Australia	Inshore waters/ inverse estuary	2013 – 2015 (2 years)	Non-calves (i.e. adults + juveniles)	15.2 ± 6.8<br>(Females = 14.7 ± 7.0)<br>(Males = 15.6 ± 6.6)	Photo-identification. Kernel (95% UD) interpolation with barriers	*This study*
Tursiops truncatus	North Inlet-Winyah Bay estuarine system, South Carolina, USA	Inshore waters/ estuary	2011 – 2012 (1 year)	Not specified	139.2	Photo-identification. Kernel (95% UD), removing landmass after calculations	(Brusa, Young, & Swanson, 2016)
Tursiops truncatus	Calibogue Sound, South Carolina, USA	Inshore waters/ estuary	1994 – 1998 (4 years)	Not specified	51.3 ± 19.1<br>(Females = 63.4 ± 28.7)<br>(Males = 51.1 ± 5.9)	Photo-identification. Adaptive Kernel (95% UD)	(Gubbins, 2002)
Tursiops truncatus	Sarasota, Florida, USA	Inshore waters	1993 – 2000 (7 years)	Adults	(Paired males = 162.6 ± 24.21)<br>(Unpaired males = 72.11 ± 24.37)	Photo-identification. Kernel (95% UD), removing landmass after calculations	(Owen, Wells, & Hofmann, 2002)
Tursiops truncatus	Sarasota, Florida, USA	Inshore waters	2005 – 2008 (3 years)	Juveniles	64.7 ± 52.6	Photo-identification. Kernel (95% UD), removing landmass after calculations	(McHugh, Allen, Barleycorn, & Wells, 2011)
Tursiops truncatus	Indian River Lagoon, Florida, USA	Inshore waters/ estuary	1997 – 2007 (10 years)	Adults	(Female with calves = 76.50 ± 10.20)<br>(Females without calves = 97.00 ± 11.50)	Photo-identification. Kernel (95% UD)	(Gibson, Howells, Lambert, Mazzoil, & Richmond, 2013)
Tursiops truncatus	Barataria Bay, Louisiana, USA	Inshore waters	2011 – 2014 (3 years)	Not specified	Females = 43.2 ± 27.55 *	Satellite tagging. Kernel (95% UD) interpolation	(Wells et al., 2017)

				(adults + subadults)	Males = 69.4 ± 30.79 *	with barriers, removing landmass after calculations	
Tursiops aduncus	Bunbury, Western Australia, Australia	Inshore waters/ estuary and coastal waters	2007 – 2013 (6 years)	Adults	Females = 65.6 ± 30.9 * Males = 94.8 ± 48.15 *	Photo-identification. Kernel (95% UD) interpolation with barriers	(Sprogis, Raudino, Rankin, MacLeod, & Bejder, 2016)
Sotalia guianensis	Cananéia estuary, São Paulo, Brazil	Inshore waters/ estuary	2000 – 2010 (10 years)	Not specified (calves to adults)	13.5 ± 13.8	Photo-identification. Kernel (95% UD), removing landmass after calculations	(de Faria Oshima & de Oliveira Santos, 2016)
Sotalia flluviatilis	Baía Norte, Santa Catarina, Brazil	Inshore waters	1996 – 2002 (6 years)	Adults + calves/juveniles	15.22 ± 0.66 (Females = 15.91 ± 1.2) (Males = 14.09 ± 1.1)	Photo-identification. Kernel (95% UD), removing landmass after calculations	(Flores & Bazzalo, 2004)

*Indicates statistically significant sex-specific differences in the size of representative ranges.

References

Brusa, J. L., Young, R. F., & Swanson, T. (2016). Abundance, Ranging Patterns, and Social Behavior of Bottlenose Dolphins (*Tursiops truncatus*) in an Estuarine Terminus. *Aquatic Mammals, 42*(1), 109-121. doi: 10.1578/AM.42.1.2016.109

de Faria Oshima, J. E., & de Oliveira Santos, M. C. (2016). Guiana dolphin home range analysis based on 11 years of photo-identification research in a tropical estuary. *Journal of Mammalogy, 97*(2), 599–610. doi: 10.1093/jmammal/gyv207

Flores, P. A., & Bazzalo, M. (2004). Home ranges and movement patterns of the marine tucuxi dolphin, *Sotalia fluviatilis*, in Baía Norte, southern Brazil. *Latin American Journal of Aquatic Mammals, 3*(1), 37-52. doi: 10.5597/lajam00047

Gibson, Q. A., Howells, E. M., Lambert, J. D., Mazzoil, M. M., & Richmond, J. P. (2013). The ranging patterns of female bottlenose dolphins with respect to reproductive status: Testing the concept of nursery areas. *Journal of Experimental Marine Biology and Ecology, 445*(0), 53-60. doi: 10.1016/j.jembe.2013.03.020

Gubbins, C. (2002). Use of home ranges by resident bottlenose dolphins (*Tursiops truncatus*) in a South Carolina estuary. *Journal of Mammalogy, 83*(1), 178-187. doi: 10.1093/jmammal/83.1.178

McHugh, K. A., Allen, J. B., Barleycorn, A. A., & Wells, R. S. (2011). Natal philopatry, ranging behavior, and habitat selection of juvenile bottlenose dolphins in Sarasota Bay, Florida. *Journal of Mammalogy, 92*(6), 1298-1313. doi: 10.1644/11-MAMM-A-026.1

Owen, E. C., Wells, R. S., & Hofmann, S. (2002). Ranging and association patterns of paired and unpaired adult male Atlantic bottlenose dolphins, *Tursiops truncatus*, in Sarasota, Florida, provide no evidence for alternative male strategies. *Canadian journal of zoology, 80*(12), 2072-2089. doi: 10.1139/z02-195

Sprogis, K. R., Raudino, H. C., Rankin, R., MacLeod, C. D., & Bejder, L. (2016). Home range size of adult Indo-Pacific bottlenose dolphins (*Tursiops aduncus*) in a coastal and estuarine system is habitat and sex-specific. *Marine Mammal Science, 32,* 287-308. doi: 10.1111/mms.12260

Wells, R. S., Schwacke, L. H., Rowles, T. K., Balmer, B. C., Zolman, E., Speakman, T., . . . Wilkinson, K. A. (2017). Ranging patterns of common bottlenose dolphins *Tursiops truncatus* in Barataria Bay, Louisiana, following the Deepwater Horizon oil spill. *Endangered Species Research, 33,* 159-180. doi: 10.3354/esr00732
